# Silver nanowires decorated with silver nanoparticles for low-haze flexible transparent conductive films

**DOI:** 10.1038/srep16371

**Published:** 2015-11-17

**Authors:** Mini Mol Menamparambath, C. Muhammed Ajmal, Kwang Hee Kim, Daejin Yang, Jongwook Roh, Hyeon Cheol Park, Chan Kwak, Jae-Young Choi, Seunghyun Baik

**Affiliations:** 1 School of Mechanical Engineering, Sungkyunkwan University, Suwon 440-746, Republic of Korea; 2 Department of Energy Science, Sungkyunkwan University, Suwon 440-746, Republic of Korea; 3 Samsung Advanced Institute of Technology, Samsung Electronics, Suwon 443-803, Republic of Korea; 4 School of Advanced Materials Science & Engineering, Sungkyunkwan University, Suwon 440-746, Republic of Korea; 5 Center for Integrated Nanostructure Physics, Institute for Basic Science (IBS), Sungkyunkwan University, Suwon 440-746, Republic of Korea

## Abstract

Silver nanowires have attracted much attention for use in flexible transparent conductive films (TCFs) due to their low sheet resistance and flexibility. However, the haze was too high for replacing indium-tin-oxide in high-quality display devices. Herein, we report flexible TCFs, which were prepared using a scalable bar-coating method, with a low sheet resistance (24.1 Ω/sq at 96.4% transmittance) and a haze (1.04%) that is comparable to that of indium-tin-oxide TCFs. To decrease the haze and maintain a low sheet resistance, small diameter silver nanowires (~20 nm) were functionalized with low-temperature surface-sintering silver nanoparticles (~5 nm) using bifunctional cysteamine. The silver nanowire-nanoparticle ink stability was excellent. The sheet resistance of the TCFs was decreased by 29.5% (from 34.2 to 24.1 Ω/sq) due to the functionalization at a low curing temperature of 85 °C. The TCFs were highly flexible and maintained their stability for more than 2 months and 10,000 bending cycles after coating with a protective layer.

There is tremendous interest in transparent conductive films (TCFs) for applications in display devices^1^,^2^, touch sensors^3^, solar cells^4^,^5^, organic photovoltaic devices^6^ and light-emitting diodes^7^. Thin films of indium-tin-oxide (ITO) exhibit excellent optoelectronic properties but brittleness impedes their application for future flexible and stretchable devices. Alternative materials to ITO, such as conducting polymers^8^, carbon nanotubes^9^,^10^, and graphene^1^, exhibit greatly improved flexibility. However, their optoelectronic properties are limited by low conductivities even after chemical doping^1^,^10^ or patterning^9^,^11^. Other alternatives consist of metal-based TCFs including metal/metal alloy grids, nanotroughs, and nanowires that exhibit excellent sheet resistance at high transmittance due to the high conductivity of metals^2^,^4^,^7^,^12^,^13^,^14^,^15^.

For real application of TCFs in display devices, one essential property is haze, which is derived from light scattering by conductive components. Haze is defined as the ratio of the difference in the total and specular transmittance to the total transmittance^16^. A large haze leads to blurriness, which becomes problematic for display devices and touch screens where clear visibility is required^17^. Therefore, studies of the haze property of TCFs have been recently conducted by various groups^14^,^15^,^18^,^19^,^20^. The haze of ITO TCFs is typically low (i.e., approximately ~1%)^14^,^15^,^18^,^19^,^20^.

Among the metal-based TCF candidates, silver nanowires (Ag NWs) are the most promising materials with respect to haze because other metal grids^12^ and metal nanotroughs^13^ have a dimension of 45 ~ 400 nm. Haze increases exponentially as the diameter of the structure increases^17^. The haze of Ag NW TCFs was more than 10% in a previously reported study^15^. The haze was also large in other studies when the sheet resistance of TCFs prepared by scalable methods was less than 30 Ω/sq (3.4% at 24 Ω/sq^14^; 4.9% at 20 Ω/sq^19^). While maintaining the similar transmittance of Ag NW TCFs, Ag NWs with a smaller diameter (~60 nm) exhibited a lower haze (12.5% at 45 Ω/sq) than films with larger diameter Ag NWs (~150 nm, haze = 31% at 35 Ω/sq) at 550 nm^18^. The trade-off between sheet resistance and haze makes the design of Ag NW TCFs challenging. For example, at a given transmittance, more nanowires are required as the diameter decreases, resulting in an increase in network junctions and sheet resistance in the TCFs.

To achieve a low sheet resistance while retaining a low haze and high transmittance for a film, resistance of the network junctions between Ag NWs should be decreased. For Ag NWs, much effort has been expended to further reduce the contact resistance at overlapping junctions using thermal (140–200 °C)^4^,^21^, mechanical^15^,^22^, and plasmonic^23^ methods. Blending with other materials^15^,^24^ or site-selective plasmon-induced chemical growth of Ag nanoparticles (NPs) after the formation of Ag NW junctions by spin coating have also been explored^25^.

Herein, we report low-temperature surface-sintering Ag NP-NW hybrid structures that enable Ag NWs to easily weld at a low temperature of ~85 °C, which is essential for applications of Ag NWs on various flexible and stretchable plastic substrates. Ag NWs (~20 nm) were decorated with Ag NPs (~5 nm) using bi-functional cysteamine as a linker. The dispersion was stable in a water-ethanol co-solvent. The nanoscale size (~5 nm) of the Ag NPs enabled effective joining of the Ag NWs at a low curing temperature (~85 °C) while maintaining a high transmittance (96.4%) and low haze (1.04%) for TCFs fabricated using a simple scalable bar-coating technology. The sheet resistance was decreased by 29.5% (from 34.2 to 24.1 Ω/sq) due to Ag NP functionalization. The low haze of the TCFs enables applications in display devices, and the low sheet resistance at high transmittance opens up a variety of application fields. The TCFs were highly flexible and maintained stability for more than 2 months and 10,000 bending cycles after coating with a protective layer.

## Results

**Figure 1:**
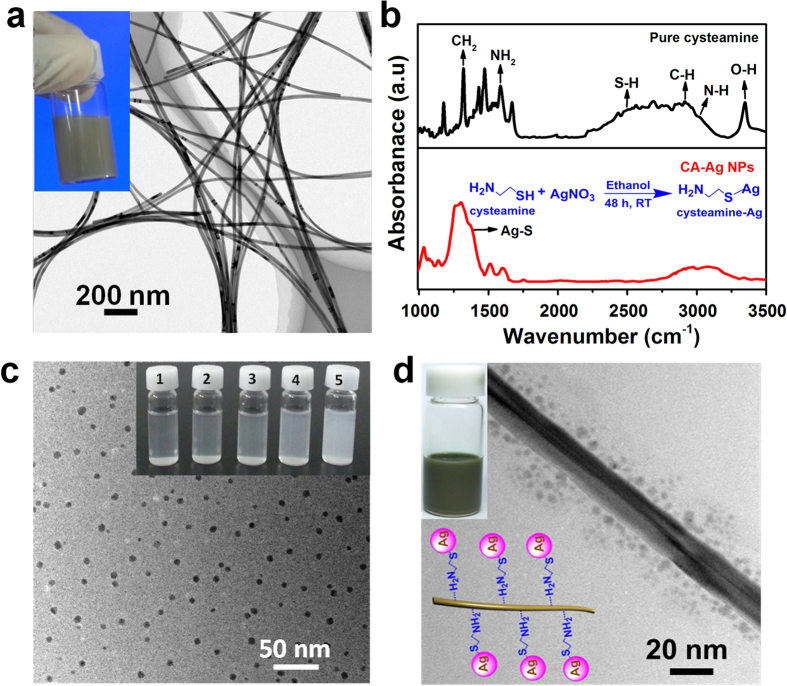
Synthesis of CA-Ag NP/Ag NWs. (**a**) HRTEM image of Ag NWs. The inset shows an aqueous suspension of Ag NWs (0.5 wt%). (**b**) FTIR spectra of pure cysteamine and CA-Ag NPs (cysteamine concentration = 6 × 10^−5^ moles). (**c**) HRTEM image of CA-Ag NPs (cysteamine concentration = 6 × 10^−5^ moles). The inset images show CA-Ag NPs (cysteamine concentration = (1) 1.5 × 10^−5^, (2) 3 × 10^−5^, (3) 6 × 10^−5^, (4) 1.2 × 10^−4^, (5) 2.4 × 10^−4^ moles) dispersed in ethanol. (**d**) HRTEM image of CA-Ag NP/Ag NWs (cysteamine concentration = 1.5 × 10^−5^ moles). The stable dispersion of CA-Ag NP/Ag NWs in an ethanol-water co-solvent is shown in the inset.

Figure 1a shows a high-resolution transmission electron microscopy (HRTEM) image of the Ag NWs (AIDEN Co. Ltd) synthesized using the polyol process with polyvinylpyrrolidone (PVP) as a stabilizer. Additional HRTEM, scanning electron microscopy (SEM), and energy-dispersive X-ray spectroscopy (EDX) analyses of the Ag NWs are provided in Supplementary Fig. S1. The average diameter, length, and aspect ratio of the Ag NWs were ~20 nm, ~18 μm, and 900, respectively. The long length (*L*) results in electrical percolation at a small critical number density of Ag NWs (*N*_*c*_), as demonstrated by Monte Carlo simulations (*N*_*c*_*L*^2^ = 5.71)^26^. The small diameter of the Ag NWs is favourable for decreasing the haze due to the decreased scattering cross-section, which will be discussed later^17^,^27^. The high aspect ratio (~900) reduces the number of Ag NW contacts and the contact resistance at a given area fraction of the film covered by Ag NWs^17^. The suspension of Ag NWs in water was stable for several months (Fig. 1a inset).

Cysteamine-functionalized Ag nanoparticles (CA-Ag NPs) were designed to attach the Ag NPs to the surface of Ag NWs. The bi-functional cysteamine contains -SH and -NH_2_ groups that can chemically interact with metal atoms^28^. Similar to benzyl mercaptan-based Ag NP functionalization^29^, the -SH moiety reacts with the Ag^+^ ion, creating a Ag-S bond upon reaction with AgNO_3_, as shown in the Fourier transform infrared (FTIR) spectra (Fig. 1b). Detailed vibrational mode assignment is provided in Supplementary Table S1. The characteristic S-H stretching vibration^30^ at 2497 cm^−1^ was absent for the CA-Ag NPs, and a new strong peak located at 1372 cm^−1^ corresponded to Ag NP interaction with the capping agent (Ag-S)^31^. This result indicated that the substitution reaction of Ag with the thiol group occurred. The intensities of the other peaks were small for CA-Ag NPs due to the low concentration of cysteamine (6 × 10^−5^ moles). See the Methods section for detailed synthesis conditions.

Figure 1c shows the HRTEM image of the CA-Ag NPs, which have an average diameter of ~5 nm. The diameter distribution and EDX analyses of the CA-Ag NPs are provided in Supplementary Fig. S2. The small size of the CA-Ag NPs resulted in TCFs with a low haze as will be discussed shortly. As shown in inset images in Fig. 1c, the concentration of CA-Ag NPs dispersed in ethanol increased as the concentration of cysteamine increased from 1.5 × 10^−5^ to 2.4 × 10^−4^ moles because the concentration of AgNO_3_ was maintained at a greater 2 × 10^−2^ moles. The optimized concentration of cysteamine was 6 × 10^−5^ moles, as discussed below. The relative weight ratio between cysteamine and the Ag NPs was determined to be 38:62% by thermogravimetric (TGA) analysis, and this value was close to the theoretical value (see Supplementary Fig. S3).

Ag NWs decorated with CA-Ag NPs (CA-Ag NP/Ag NWs) were obtained by mixing Ag NW and CA-Ag NP suspensions. The -NH_2_ group of cysteamine facilitates anchoring of CA-Ag NPs on the surface of Ag NWs through surface polymerization^28^,^32^. Uniform adsorption of CA-Ag NPs around the Ag NWs is shown in Fig. 1d. The EDX analysis from scanning transmission electron microscopy confirmed that the dots (~5 nm) around the Ag NWs were Ag NPs (see Supplementary Fig. S4). A stable suspension of CA-Ag NP/Ag NWs in an ethanol-water co-solvent was obtained, as shown in the inset of Fig. 1d.

**Figure 2:**
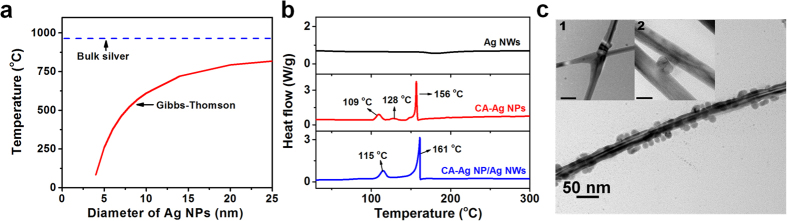
Melting and coalescence analysis of Ag NPs and CA-Ag NP/Ag NWs. (**a**) The theoretical melting temperature of spherical Ag NPs was calculated using the Gibbs-Thomson equation. The melting temperature of bulk silver is 962 °C. (**b**) DSC analysis of Ag NWs, CA-Ag NPs, and CA-Ag NP/Ag NWs. (**c**) HRTEM images of CA-Ag NP/Ag NWs heat treated at 110 °C. The scale bars in the insets correspond to 50 nm (1) and 20 nm (2).

The melting temperature of the materials decreases as the size is decreased to the nanoscale due to the change in cohesive energy of nanomaterials^33^,^34^,^35^,^36^. Less energy is required for surface atoms to move because they have fewer bonds with each other at the free surface^33^. In contrast, the atoms in the bulk are evenly surrounded^35^. The melting temperature (*T*_*m*_) of spherical particles can be calculated as a function of the diameter (*d*_*s*_) using the Gibbs-Thomson equation^33^,^36^.


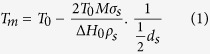


where *M, ΔH*_0_, and *ρ*_*s*_ are the molar mass (107.87 g/mol), enthalpy of melting (11.28 kJ/mol) and mass density (10.49 g/cm^3^) of silver, respectively. *T*_0_ is the melting temperature of bulk silver (962 °C). *σ*_*s*_ is the surface energy density (0.78 J/m^2^) of Ag NPs (diameter range = 2 ~ 10 nm)^37^. As shown in Fig. 2a, *T*_*m*_ decreases rapidly when *d*_*s*_ is smaller than 20 nm, resulting in *T*_*m*_ = 250 °C at *d*_*s*_ = 5 nm. The coalescence of particles occurs even at a temperature lower than *T*_*m*_, which is experimentally characterized by differential scanning calorimetry (DSC) (Fig. 2b)^38^. Exothermic peaks are observed as the energy level of particles decreases due to coalescence of the particles^34^. No distinct exothermic peak was observed for the Ag NWs, indicating the thermal stability of the Ag NWs that were synthesized using the polyol method. In contrast, three exothermic peaks were observed at 109, 128 and 156 °C for the CA-Ag NPs due to the multi-step coalescence of the CA-Ag NPs with a size distribution (see Supplementary Fig. S2). Two exothermic peaks were observed for the CA Ag-NP/Ag NWs at higher temperatures (115 and 161 °C). The active coalescence among Ag NPs was hindered by Ag NWs increasing coalescence temperatures. Figure 2c shows the HRTEM images of CA-Ag NP/Ag NWs heat-treated at 110 °C. The Ag NPs attached to the surface of the Ag NWs coalesced, increasing their size to 10~20 nm. Ag NWs were joined when Ag NPs were present at junctions of Ag NWs or between adjacent Ag NWs (Fig. 2c insets). A higher heat-treatment temperature (≥140 °C) is typically employed for Ag NW-based TCFs^4^,^21^. A low heat-treatment temperature enables energy efficient fabrication of TCFs without degradation of the flexible polymer substrates, which is favourable in the flexible electronics industry.

**Figure 3:**
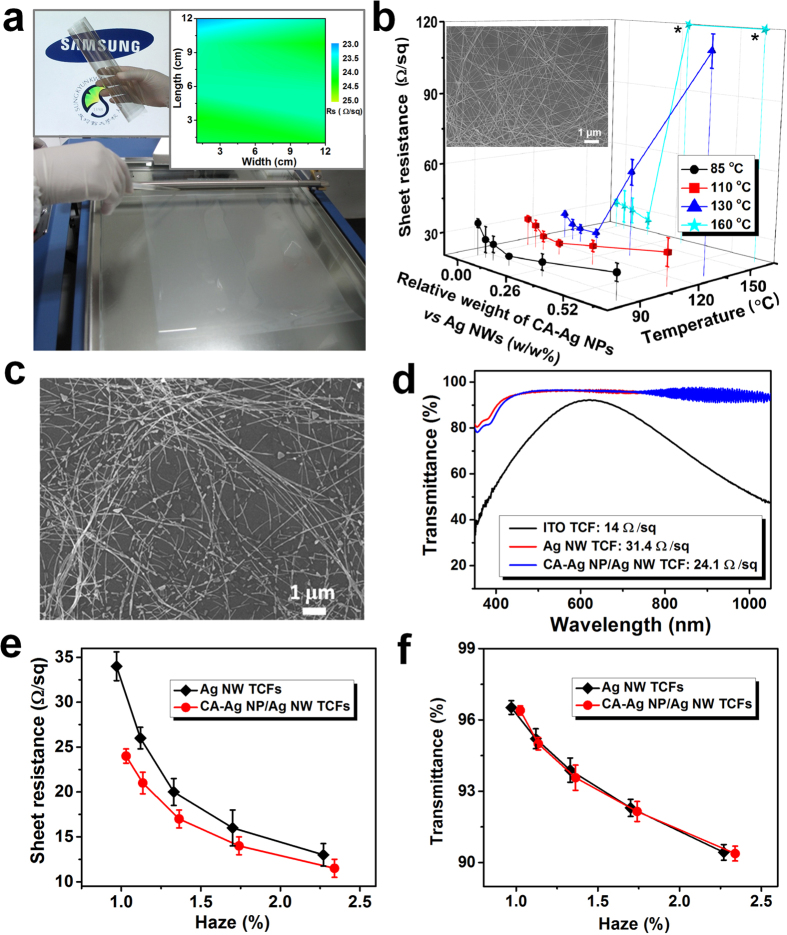
Electrical and optical properties of TCFs. (**a**) Fabrication of CA-Ag NP/Ag NW TCFs on PC substrates (21 × 29.7 cm^2^). The inset images show the flexibility and area map for the sheet resistance of TCFs. The logos used in the figure were reproduced with permission from Samsung electronics and Sungkyunkwan University. (**b**) Sheet resistances of TCFs are shown as a function of the heat-treatment temperature and relative weight concentration of CA-Ag NPs and Ag NWs. The concentration of Ag NWs was fixed at 41.3 wt%. An SEM image of CA-Ag NP/Ag NW TCF cured at 85 °C (CA-Ag NP/Ag NW = 0.16 w/w%) is provided in the inset. (**c**) SEM image of CA-Ag NP/Ag NWs on a Si/SiO_2_ substrate. The relative concentration of CA-Ag NPs over Ag NWs was 0.16 w/w%, and the curing temperature was 160 °C. (**d**) Transmittance spectra of ITO, Ag NW, and CA-Ag NP/Ag NW TCFs. (**e,f**) Sheet resistance and total transmittance of Ag NW and CA-Ag NP/Ag NW TCFs are shown as a function of the haze. The Ag NW concentration was varied from 41.3 to 70.6 wt%. The relative concentration of CA-Ag NPs over Ag NWs was fixed at 0.16 w/w% for CA-Ag NP/Ag NWs. The curing temperature was 85 °C.

As shown in Fig. 3a, flexible TCFs were fabricated using CA-Ag NP/Ag NWs (see Methods for details). Briefly, the CA-Ag NP/Ag NW solution was diluted using ethanol (16.23 wt%), water (23.86%), and a binder (hydroxypropylmethyl cellulose in water, 4.15 wt%) followed by bar-coating on polycarbonate (PC) substrates. The PC substrate was flexible with a small root-mean-square (RMS) roughness of 3.2 nm (see Supplementary Fig. S5). The ethanol-water co-solvent with the binder improved the dispersion and adhesion of the CA-Ag NP/Ag NWs on the PC substrate. The TCF was flexible, as shown in Fig. 3a inset. The area map of the sheet resistance is also provided and demonstrates the uniformity of the TCF (curing temperature = 85 °C, relative concentration of CA-Ag NPs over Ag NWs = 0.16 w/w%). This simple solution-based coating technology is another advantage for decreasing TCF fabrication costs compared to vapour-phase sputtering or multi-step processes^17^.

Figure 3b shows the sheet resistances of the TCFs cured at different temperatures (85, 110, 130, and 160 °C). The concentration of Ag NWs was maintained at 41.3 wt%. The relative weight concentration between CA-Ag NPs and Ag NWs was varied between 0 and 0.65 w/w%. The variation in the total concentration of CA-Ag NP/Ag NWs was small (41.30 ~ 41.31%). The sheet resistance, transmittance, and haze of the TCFs for the data shown in Fig. 3b are provided in Supplementary Table S2. The transmittance and haze of the TCFs were 95 ~ 96% and 0.9 ~ 1.2%, respectively, except for the films cured at high temperatures (130 and 160 °C) with high relative concentrations of CA-Ag NPs (0.32 and 0.65 wt%). The sheet resistance of the TCFs prepared using pure Ag NWs (relative concentration of CA-Ag NPs = 0 w/w%) increased from 31.4 to 34.2 Ω/sq as the curing temperature decreased from 130 to 85 °C. At a low curing temperature of 85 °C, the sheet resistance decreased from 34.2 to 24.1 Ω/sq as the relative concentration of CA-Ag NPs increased from 0 to 0.16 w/w%. The corresponding transmittance and haze were 96.4 and 1.04%. An SEM image of a uniformly distributed network of CA-Ag NP/Ag NWs is shown in the inset of Fig. 3b. The low sheet resistance and haze at a low curing temperature was achieved due to the active coalescing activity of the Ag NPs. The low sheet resistance was obtained at a curing temperature that was even lower than the sintering temperature (115 °C) observed from DSC analysis, which was most likely due to the active surface atoms of Ag NPs^34^. A similar trend was observed at a curing temperature of 110 °C. The minimum sheet resistance was 24.9 Ω/sq (transmittance = 96.5%, haze = 1.04%) at a relative CA-Ag NPs concentration of 0.16 w/w%. However, the sheet resistance was very large (≥56 Ω/sq) when the curing temperature was ≥130 °C at high relative weight concentrations of CA-Ag NPs (≥0.32 w/w%). The haze of the TCFs also increased (≥1.39%). To investigate the mechanism of achieving high sheet resistance and haze, CA-Ag NP/Ag NWs were heat treated at 160 °C on a Si/SiO_2_ substrate (Fig. 3c). Surprisingly, numerous broken short Ag NWs were observed. In contrast, the pristine length was preserved when pure Ag NWs were heat treated at 160 °C (see Supplementary Fig. S6a). The cutting of wires was also observed when pure cysteamine was added to pure Ag NWs, indicating the important role of cysteamine (Supplementary Fig. S6b). The melting temperature of pure cysteamine was ~97 °C (see Supplementary Fig. S7), and the liquid phase of cysteamine may have actively interfered with and removed PVP around the Ag NWs. The PVP was present on the surface of the Ag NWs synthesized using the polyol method, which improved the dispersion and stability of the Ag NWs. The removal of PVP around the Ag NWs may increase the instability of the Ag NWs during the curing process even though the precise mechanism needs to be further investigated. The cutting of Ag NWs also increased the haze of the TCFs, as shown in Supplementary Fig. S8.

As shown in Fig. 3d, the transmittance of ITO (Solaronix, PETITO175-14, 14 Ω/sq) decreased in the near-infrared region due to plasmon resonance^39^. However, the transmittance of the TCFs prepared using Ag NWs and CA-Ag NP/Ag NWs did not change over a wide range of wavelengths. Figure 3e shows the sheet resistance of the TCFs prepared using pure Ag NWs as a function of the haze. The sheet resistance decreased from 34.2 to 11.1 Ω/sq as the concentration of Ag NWs increased from 41.3 to 70.6 wt%. The corresponding haze increased from 0.96 to 2.3%. The addition of CA-Ag NPs to the Ag NWs (i.e., CA-Ag NP/Ag NWs) systematically decreased the sheet resistance without significantly altering the haze. The sheet resistance decreased by 29.5% (from 34.2 to 24.1 Ω/sq) at a low haze of 1.04% and a high transmittance of 96.4%, demonstrating enhanced joining of the Ag NWs with the Ag NPs. The sheet resistance further decreased and the haze increased as the concentration of CA-Ag NP/Ag NWs increased. As shown in Fig. 3f, the transmittance decreased as the haze increased upon addition of Ag NWs or CA-Ag NP/Ag NWs. The haze increased roughly linearly with the area fraction of the film covered by Ag NWs^17^. The UV-Vis-NIR spectra of the TCFs shown in Fig. 3e,f were also provided in Supplementary Fig. S9. The interpolation of the data in Fig. 3e,f suggests that a greater haze (~1.2%) and a lower transmittance (~94.6%) would be required to obtain a sheet resistance of 24.1 Ω/sq with pure Ag NWs. This result clearly demonstrates the advantages of employing CA-Ag NP/Ag NW ink for TCFs. The simple CA-Ag NP/Ag NW ink could decrease the sheet resistance of TCFs cured at low temperatures (85 °C) while maintaining high transmittance and low haze.

**Figure 4:**
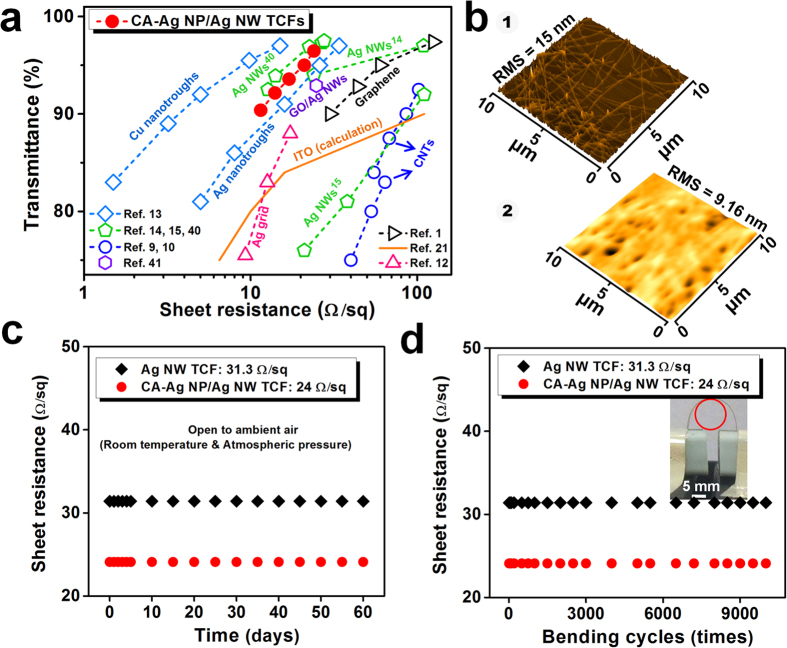
Performance comparison, stability, and flexibility of TCFs made using CA-Ag NP/Ag NWs. (**a**) Comparison of sheet resistance and transmittance of CA-Ag NP/Ag NW TCFs (solid symbols) to the results obtained from the literature (open symbols). The Ag NW concentration was varied from 41.3 to 70.6 wt% (CA-Ag NPs/Ag NWs = 0.16 w/w%). The curing temperature was 85 °C. The analysis does not include the transmittance of supporting substrates except for the data in ref. 41. (**b**) AFM images of CA-Ag NP/Ag NW TCFs before (1) and after (2) the over-layer coating. (**c**) Sheet resistance stability of Ag NW and CA-Ag NP/Ag NW TCFs exposed to ambient air for 2 months. (**d**) Bending cyclability of Ag NW and CA-Ag NP/Ag NW TCFs. The minimum bending radius was 5 mm.

The sheet resistances of the CA-Ag NP/Ag NW TCFs (filled circle symbols) are compared with control data from the literature (open symbols), as shown in Fig. 4a. TCFs in the literature with sheet resistance values greater than 150 Ω/sq were not included. Chemical doping or patterning was typically employed for carbon nanotube TCFs to decrease the sheet resistance^9^,^10^. However, the resistance was typically greater than the theoretical prediction for ITO^21^. The chemically doped graphene TCFs provided smaller sheet resistances than carbon nanotube TCFs at equivalent transmittances^1^. The Ag grid TCFs patterned using expensive electron beam lithography provided smaller sheet resistances compared to those of graphene TCFs even though the transmittance was lower^12^. The sheet resistance and transmittance of Ag NW TCFs were highly dependent on the length or aspect ratio of the Ag NWs^14^,^15^,^40^. The long Ag NWs (diameter = ~91 nm, length = 20~230 μm)^14^ provided higher transmittances than short Ag NWs (diameter = 40~100 nm, length = ~10 μm)^15^ at equivalent sheet resistances. However, the haze was high (1.6% at 109 Ω/sq and 3.4% at 24 Ω/sq) for the large Ag NW TCFs^14^. The TCFs prepared by spin-coating of high aspect ratio Ag NWs (average diameter = 25 nm, length = a few tens of micrometres) onto PET films provided a similar or slightly better performance compared to that of CA-Ag NP/Ag NW TCFs^40^. However, the spin-coating process is not scalable for mass production^17^, and systematic uniform properties are difficult to maintain over a large area as a function of the process parameter. Indeed, spin-coating (2000 rpm, 40 s) of CA-Ag NP/Ag NWs (CA-Ag NPs/Ag NWs = 0.16 w/w%) on a polyethyleneterephthalate (PET) film (5 × 5 cm^2^) produced greater variation (average sheet resistance = 27.5 Ω/sq, standard deviation = 5.10 Ω/sq, average transmittance = 97.2%) compared to that (mean sheet resistance = 24.1 Ω/sq, standard deviation = 0.82 Ω/sq, average transmittance = 96.4%) for the bar-coated sample (see Supplementary Table S2) at the same curing temperature (85 °C). The over-coating of Ag NWs with graphene oxide (GO) nanosheets decreased the sheet resistance from ~30.3 to ~24.8 Ω/sq at a transmittance of ~92.9% due to strong adhesion of the GO nanosheets to the supporting plasma-treated hydrophilic PET substrates^41^. However, the haze was high (~2.7%). The transmittance of supporting PET substrates was not separately characterized in ref. 41.

Copper nanotrough TCFs provided better performance than CA-Ag NP/Ag NW TCFs^13^. However, multi-step processes, such as electrospinning of long polymer fibres, thin metal deposition, and dissolution of template fibres, were required to synthesize the metal nanotroughs^13^. The large diameter (~400 nm) of nanotroughs may increase the extinction cross-sections^17^ and light scattering even though the precise haze was not reported in the literature^13^. A potential oxidation issue also exists for Cu nanotroughs. The properties of TCFs prepared using Ag NWs were also compared in Supplementary Table S3. The sheet resistance, transmittance, haze, fabrication methods, diameter and length of Ag NWs were compared. A low sheet resistance of 24.1 Ω/sq was obtained at a high transmittance of 96.4% and a low haze of 1.04% using the simple bar-coating technology along with stable CA-Ag NP/Ag NW ink. The performance of CA-Ag NP/Ag NW TCFs may be further improved by a post treatment, such as patterning^9^,^10^, which is beyond the scope of this work.

Ag_2_S is formed by reaction with atmospheric sulfides, and Ag reduces the conductivity and increases the scattering cross-section of Ag NWs^17^,^42^. To enhance the stability of TCFs, an ultraviolet-curable urethane-acrylate based protective layer was coated on the CA-Ag NP/Ag NWs (Fig. 4b). A uniform distribution of CA-Ag NP/Ag NWs with a RMS of 15 nm was observed prior to coating with the protective layer. The surface roughness decreased to 9.2 nm after the coating. The uniform and smooth surface maintains high optical transmission due to less scattering. Visual demonstration of the uniformity of the sheet resistance after the protective layer coating is shown in Supplementary Fig. 10. As shown in Fig. 4c, the sheet resistance of the protective layer-coated TCFs prepared using pure Ag NWs and CA-Ag NP/Ag NWs was invariant over 60 days under ambient air conditions. The sheet resistance did not change even when the TCFs went through 10,000 bending cycles with a minimum bending radius of 5 mm (Fig. 4d). This result demonstrates the excellent stability and flexibility of the TCFs after application of the protective layer.

One-dimensional Ag NWs are advantageous for achieving percolation at low concentration due to the high aspect ratio. However, the contact hinders further reduction in the sheet resistance because Ag NWs are covered by PVP during the polyol synthesis process. Zero-dimensional Ag NPs with high surface-area-to-volume ratios can undergo vigorous coalescence during curing because less energy is required to move surface atoms^33^,^34^,^35^,^36^. The one-dimensional Ag NWs are combined with zero-dimensional Ag NPs in this study to exploit the advantages of each component. Therefore, the sheet resistance of the TCFs decreases while maintaining high transmittance and low haze. This study demonstrates the potential to replace Ag NWs with CA-Ag NP/Ag NWs for TCFs because the synthesis process is simple and scalable.

In summary, Ag NPs were attached to the surface of Ag NWs using bi-functional cysteamine. The dispersion of CA-Ag NP/Ag NWs was stable in a water-ethanol co-solvent. The nanoscale size (~5 nm) of the Ag NPs enabled effective joining with Ag NWs at a low curing temperature (~85 °C). The sheet resistance was decreased by 29.5% (from 34.2 to 24.1 Ω/sq) due to functionalization with Ag NPs, and a high transmittance of 96.4% and a low haze of 1.04% were achieved. The TCFs prepared using CA-Ag NP/Ag NWs were highly flexible and maintained their stability for more than 2 months and 10,000 bending cycles after coating with a protective layer.

## Methods

### Synthesis of CA-Ag NP/Ag NWs

CA-Ag NPs were synthesized in ethanol (Daejung, 4022-4410) using a self-assembly method. A cysteamine solution in ethanol (Sigma Aldrich, M9768, 0.1 M, 0.15–2.4 ml) was added to a silver nitrate solution in ethanol (High purity chemicals, 3692741, 0.02 M, 300 ml). The resulting whitish solution was stirred for 48 hours at room temperature to obtain well-dispersed CA-Ag NPs (average diameter = ~5 nm). In the next step, the CA-Ag NP solution was mixed with Ag NWs (AIDEN Co. Ltd., average diameter = ~20 nm, length = ~18 μm) dispersed in water (0.5 wt%, 4.15 ml) by mechanical stirring (40 rpm, 2 hours). The relative ratio between the Ag NPs and the Ag NWs was varied from 0 to 0.65 wt%. Then, the CA-Ag NP/Ag NW ink was prepared by adding a co-solvent (de-ionized water: 2.41 ml, anhydrous ethanol: 2.08 ml) and an aqueous hydroxypropylmethyl cellulose binder (Sigma Aldrich, 09965, 0.25 wt%, 0.42 ml).

### Fabrication of TCFs

The TCFs were fabricated by bar-coating (Meyer rod, RDS 10, 30 mm/sec) CA-Ag NP/Ag NW inks onto flexible PC substrates (21 × 29.7 cm^2^, transmittance = 90.1%). Then, the film was dried and heat treated (85, 110, 130, or 160 °C) for 10 minutes. For the stability tests, a urethane-acrylate based protective layer was applied using a bar-coating technique (Meyer rod, RDS 4) followed by drying in air (15 minutes) and ultraviolet curing (~800 mW/cm^2^, 2 minutes).

### Characterization

The sample morphologies were characterized by SEM (JOEL, JSM-7500F), HRTEM (JOEL, JEM-2100F), and atomic force microscopy (Veeco, diINNOVA 840-012-711). The chemical compositions of the reagents and specimens were determined using an FTIR spectrometer (Bruker, IFS-66/S). The sheet resistance was measured at 27 points on each TCF using a four-point probe surface resistivity meter (R-CHEK, RC2175). The specular transmittance was measured between 350 and 1050 nm (UV-Vis-NIR spectrophotometer UV3600). The total transmittance and haze of the TCFs were also measured using white light (Nippon Denshoku Industries Co., NDH 7000 haze meter, 400 ~ 700 nm). The transmittance of the PC substrate was not included in the total transmittance analysis. The haze was calculated as *I*_*s,2°–90°*_*/(I*_*d*_ *+ I*_*s,0°–90°*_). *I*_*s,2°-90°*_ is the forward scattered intensity between 2° and 90°, *I*_*d*_ is the direct parallel transmitted intensity, and *I*_*s,0°–90°*_ is the forward scattered intensity between 0° and 90°. The total transmittance is *I*_*d*_ *+* *I*_*s,0°–90°*._ TGA (Seiko Exstar 6000, TG/DTA6100) and DSC (Seiko Exstar 6000, DSC6100) analyses were carried out under a nitrogen atmosphere.

## Additional Information

**How to cite this article**: Mol Menamparambath, M. *et al.* Silver nanowires decorated with silver nanoparticles for low-haze flexible transparent conductive films. *Sci. Rep.* **5**, 16371; doi: 10.1038/srep16371 (2015).

## Supplementary Material

Supplementary Information
