# Supplementary material for: Silver nanowires decorated with silver nanoparticles for low-haze flexible transparent conductive films
Source: Sci Rep. 2015 Nov 17;5:16371. doi: 10.1038/srep16371 (PMC4648094; doi:10.1038/srep16371)
Supplement: Supplementary Information [file srep16371-s1.pdf]

SUPPLEMENTARY INFORMATION

Silver nanowires decorated with silver nanoparticles for low-haze flexible transparent conductive films

Mini Mol Menamparambath[1,†], C. Muhammed Ajmal[2,†], Kwang Hee Kim[3], Daejin Yang[3], Jongwook Roh[3], Hyeon Cheol Park[3], Chan Kwak[3], Jae-Young Choi[3,4,*], Seunghyun Baik[1,5,*]

[1] School of Mechanical Engineering, Sungkyunkwan University, Suwon 440-746, Republic of Korea

[2] Department of Energy Science, Sungkyunkwan University, Suwon 440-746, Republic of Korea

[3] Samsung Advanced Institute of Technology, Samsung Electronics, Suwon 443-803, Republic of Korea

[4] School of Advanced Materials Science & Engineering, Sungkyunkwan University, Suwon 440-746, Republic of Korea

[5] Center for Integrated Nanostructure Physics, Institute for Basic Science (IBS), Sungkyunkwan University, Suwon 440-746, Republic of Korea

[†] These authors contributed equally to this work.

Corresponding Authors

E-mail: jy.choi@skku.edu; sbaik@me.skku.ac.kr

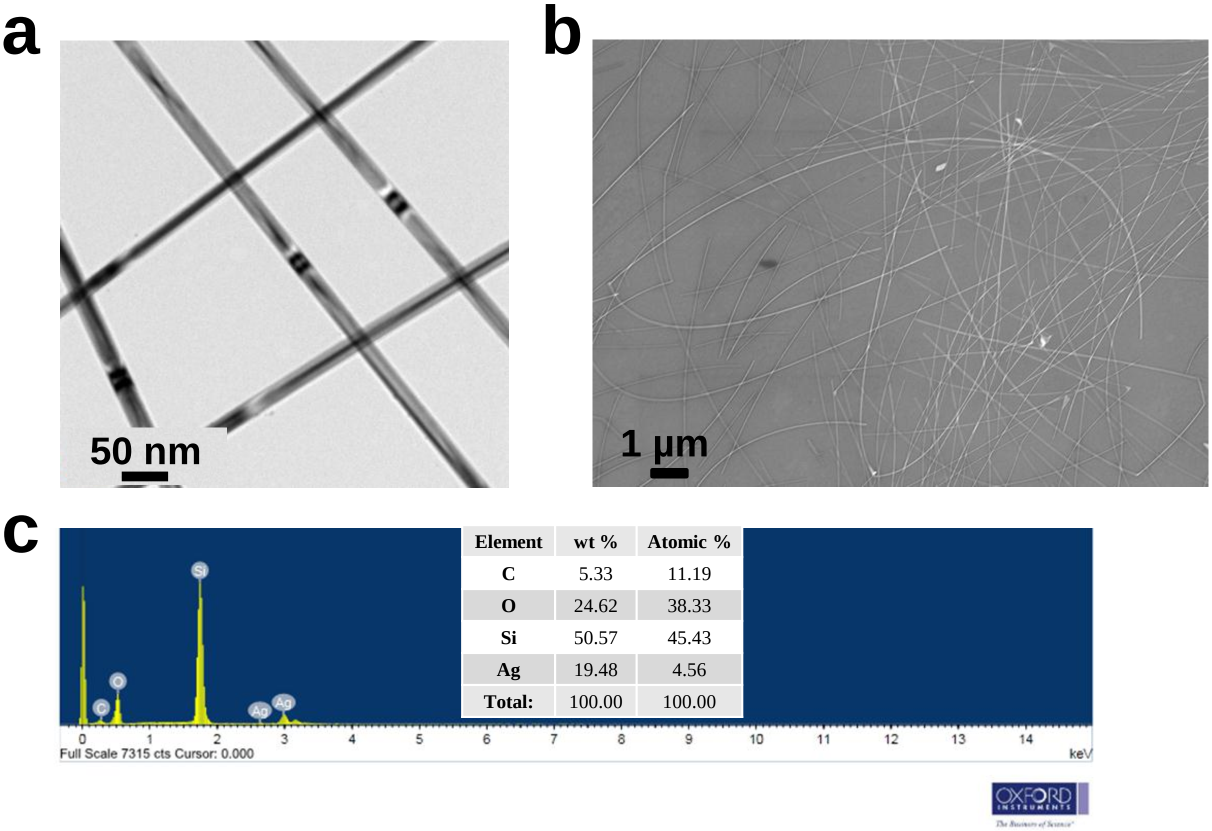

Element	wt %	Atomic %
C	5.33	11.19
O	24.62	38.33
Si	50.57	45.43
Ag	19.48	4.56
Total:	100.00	100.00

Figure S1. HRTEM (a), SEM (b), and EDX (c) analyses of Ag NWs. Carbon and oxygen peaks were observed due to the PVP covering around Ag NWs. Silicon and oxygen peaks came from the Si/SiO_2 substrate.

Vibration mode	Wavenumber (cm^{-1})
N-H stretch[s1]	3018
S-H stretch[s1]	2497
CH_2 stretch[s2]	2918, 2850
CH_2 deformation[s2]	1384–1393
C–C stretch[s2]	1200
NH_2 deformation[s3]	1587
CH_2 wagging deformation[s3]	1247
C=O stretch[s3]	1670
coupled –CH_2 deformation[s3]	1495, 1325
Ag-S (Ag NP and capping agent interaction) [s4]	1372
O-H group[s4]	3200
C-N stretch[s5]	1071
O-H group[s5]	3347

Table S1. Vibrational mode assignment in FTIR analysis.

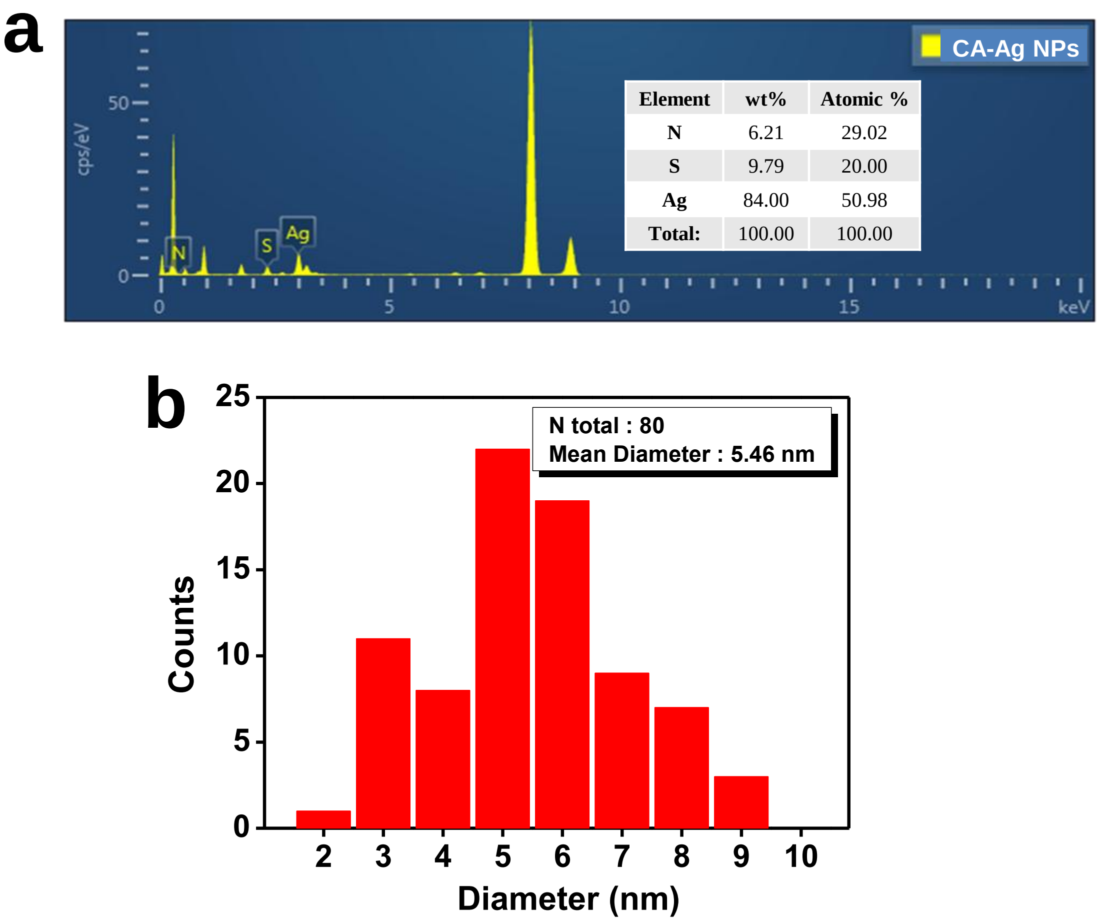

Figure S2. HRTEM analysis of CA-Ag NPs. (a) EDX analysis from scanning transmission electron microscopy (JOEL, JEM-2100F) indicated the presence of Ag, N, and S elements. Carbon was excluded in the atomic composition analysis due to the interference with a lacey carbon TEM grid. (b) Diameter distribution of CA-Ag NPs obtained from HRTEM images.

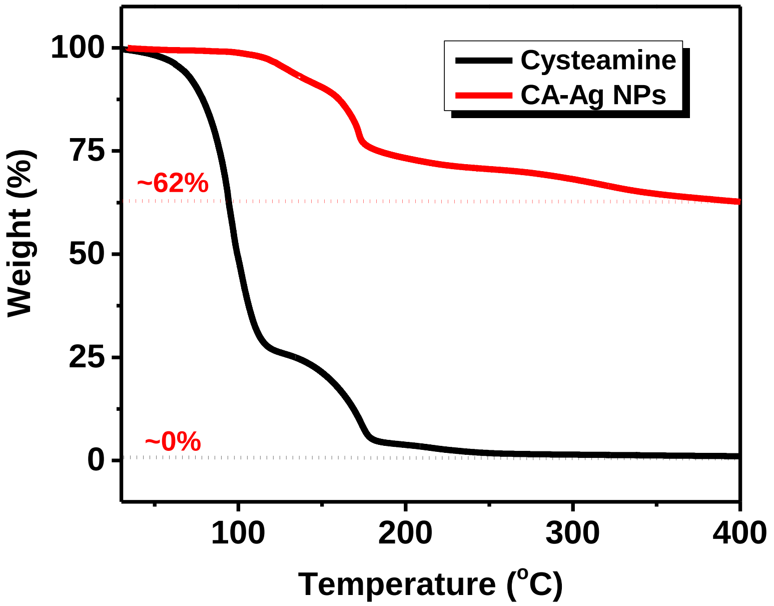

Figure S3. Thermogravimetric analysis of pure cysteamine and CA-Ag NPs. CA-Ag NP powders were firstly obtained by centrifuging suspended CA-Ag NPs in ethanol and drying in vacuum oven (room temperature) for 12 h. The thermogravimetric analysis was then carried out using dried CA-Ag NP powders. The concentrations of cysteamine and $AgNO_3$ were 6×10^{-5} and 2×10^{-2} moles in the synthesis solution of CA-Ag NPs, and the theoretical weight ratio between Ag and cysteamine was 58:42%. The remaining weight concentration of CA-Ag NPs at 400 °C was 62% from thermogravimetric analysis. The 38% weight loss corresponds to the dissociation of cysteamine. A complete dissociation of pure cysteamine was observed at 400 °C.

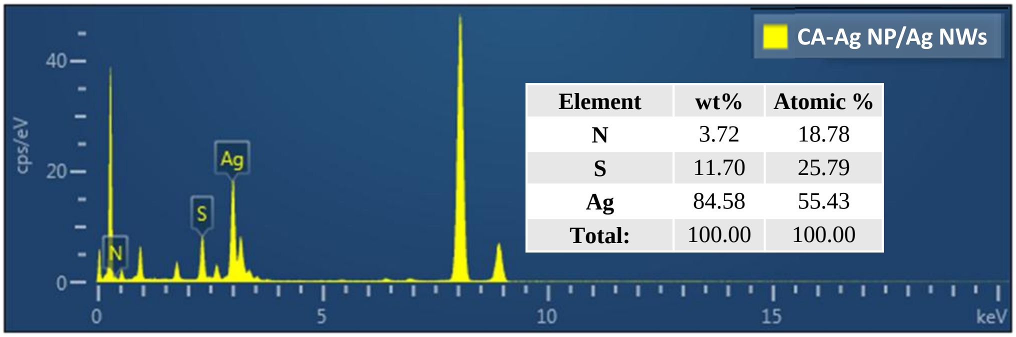

Element	wt%	Atomic %
N	3.72	18.78
S	11.70	25.79
Ag	84.58	55.43
Total:	100.00	100.00

Figure S4. **Scanning transmission electron microscopy (JOEL, JEM-2100F) analysis of CA-Ag NPs attached to Ag NWs.** EDX analysis indicated the presence of Ag, N, and S elements.

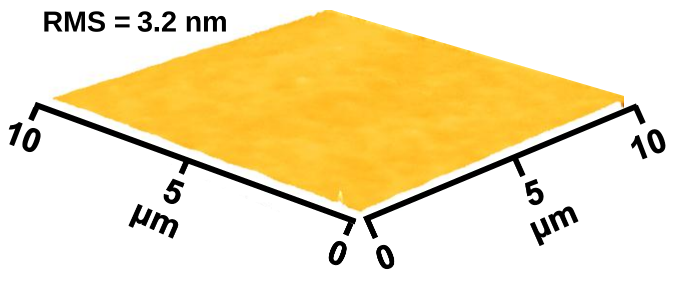

Figure S5. The surface roughness of the PC substrate measured by atomic force microscopy.

Relative weight concentration of CA-Ag NPs over Ag NWs (w/w%)	Curing Temp. (^{o}C)	Sheet resistance (Ω/sq)	Sheet resistance Std. Dev.	Transmittance (%)	Transmittance Std. Dev.	Haze (%)	Haze Std. Dev.
0	85	34.2	1.90	96.5	0.19	0.96	0.03
	110	32.0	1.44	96.6	0.35	1.04	0.03
	130	31.4	1.50	96.4	0.25	1.03	0.09
	160	32.2	1.66	96.5	0.11	1.04	0.04
0.04	85	28.4	5.68	96.5	0.15	1.09	0.04
	110	30.5	2.36	96.4	0.12	1.22	0.03
	130	27.4	2.42	96.6	0.19	1.14	0.07
	160	31.4	7.70	96.4	0.21	1.18	0.08
0.08	85	26.5	3.57	96.4	0.20	0.98	0.04
	110	26.4	2.38	96.3	0.09	1.16	0.05
	130	26.1	2.02	95.6	0.24	1.13	0.11
	160	29.5	6.03	95.2	0.15	1.20	0.12
0.16	85	24.1	0.80	96.4	0.09	1.04	0.05
	110	24.9	1.54	96.5	0.07	1.04	0.04
	130	25.6	1.81	96.1	0.25	1.17	0.28
	160	27.2	3.75	95.5	0.25	1.19	0.30
0.32	85	26.4	3.40	96.2	0.13	1.00	0.04
	110	28.0	2.43	96.0	0.23	1.00	0.08
	130	56.6	5.62	95.3	0.50	1.39	0.30
	160	Out of range	-	94.5	0.52	1.60	0.35
0.65	85	31.0	3.53	95.9	0.11	1.16	0.02
	110	34.4	5.80	96.0	0.09	1.24	0.10
	130	111.6	6.78	94.6	0.55	2.31	0.35
	160	Out of range	-	94.1	0.71	2.50	0.50

Table S2. Sheet resistance, transmittance, and haze of TCFs corresponding to the data in Fig. 3b. Sheet resistance was measured at 27 different locations of each sample. Transmittance and haze were measured at 9 different locations of each sample. The mean values and standard deviations (Std. Dev.) are provided.

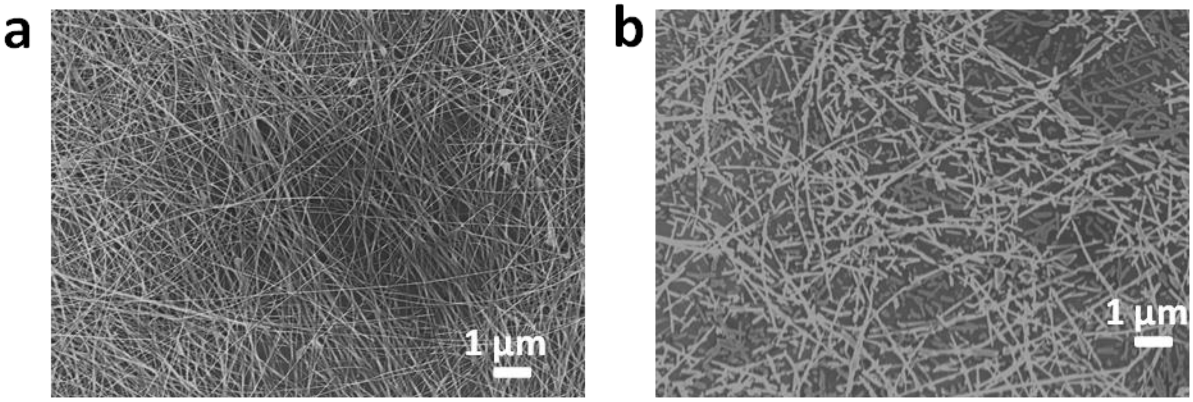

Figure S6. SEM images of pure Ag NWs (a) and cysteamine-added Ag NWs (b) heat-treated at 160 ºC on Si/SiO_2 substrates. The cysteamine concentration was 6×10^{-5} moles.

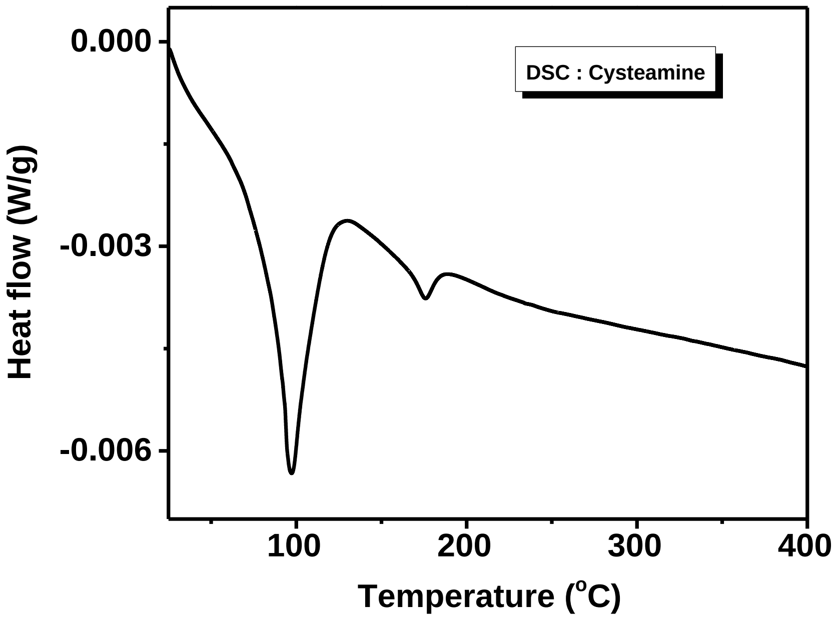

Figure S7. Differential scanning calorimetry analysis of pure cysteamine. Endothermic peaks at 97 and 175 ºC indicate melting and dissociation of cysteamine.

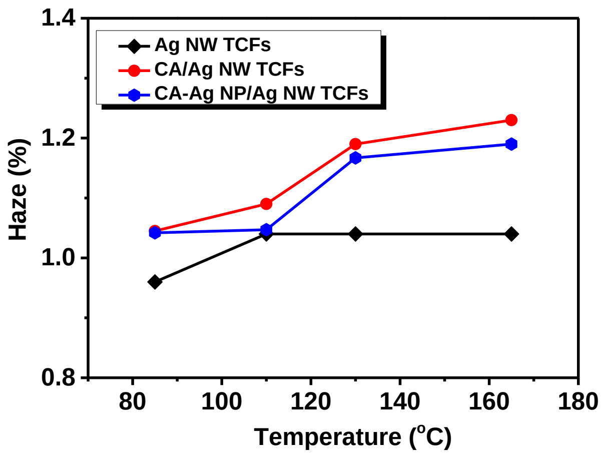

Figure S8. Haze of TCFs made using pure Ag NWs, cysteamine-added Ag NWs, and CA-Ag NP/Ag NWs cured at different temperatures. The cysteamine concentration was 6×10^{-5} moles. The relative concentration of CA-Ag NPs over Ag NWs was 0.16 w/w% for CA-Ag NP/Ag NWs.

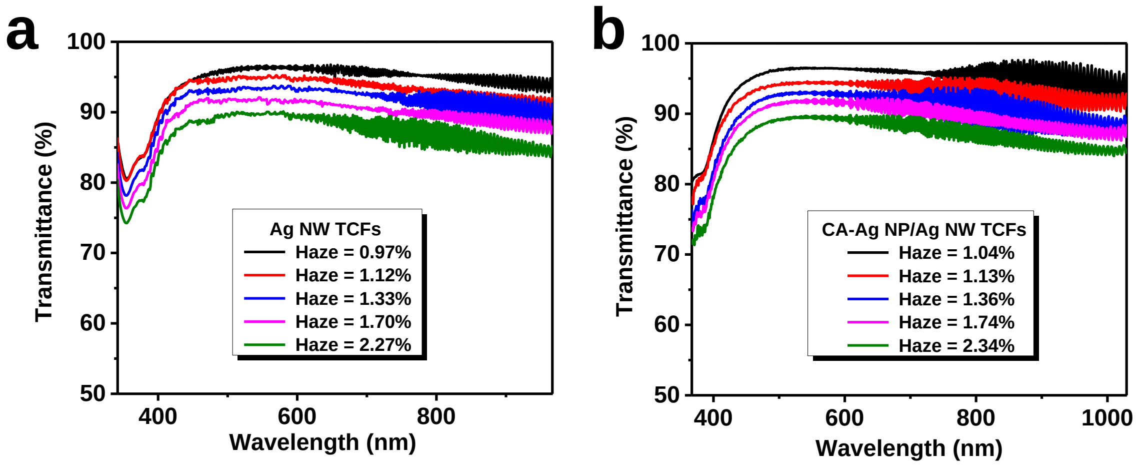

Figure S9. The UV-Vis-NIR transmittance spectra of TCFs shown in Figs. 3e and 3f. (a) Ag NW TCFs. (b) CA-Ag NP/Ag NW TCFs.

Conductive material	Sheet resistance (Ω/sq)	Transmittance (%)	Haze (%)	Ag NW Length	Ag NW Diameter	Fabrication	
						Method	Annealing condition
**CA-Ag NP/Ag NWs (This work)**	24.1<br>21.3<br>17.0<br>13.9<br>11.5	96.4<br>95.00<br>93.57<br>92.15<br>90.38	1.04<br>1.13<br>1.36<br>1.74<br>2.34	18 μm	20 nm	Bar coating	85°C for 10 min
**Ag NWs** [s6]	21<br>38<br>110	76<br>81<br>92	10	10 μm	40-100 nm	Bar coating	120 °C
**Ag NWs** [s7]	24<br>109	97<br>94	3.4<br>1.6	20-230 μm	91 nm	Drop coating	200 °C for 10 min
**Ag NWs** [s8]	24.8	92.9	2.7	NA	NA	Bar coating	140 °C for 4 min
**Ag NWs** [s9]	12.7<br>14.1<br>22.5<br>26.8<br>27.6	92.5<br>93.9<br>96.9<br>97.3<br>97.5	NA	Few tens of μm	25 nm	Spin coating	120 °C for 5 min
**Ag NWs** [s10]	59<br>20<br>11	97<br>92.1<br>87	2.5<br>4.9<br>7.5	13.5 μm	62.5 nm	Electrostatic spray deposition	120 °C for 8 hours
**Ag NWs** [s11]	35<br>45	74.8<br>77	31<br>12.5	20 μm<br>25 μm	150 nm<br>60 nm	Vacuum filtration	Room temperature

Table S3. The properties of TCFs made using Ag NWs were compared.

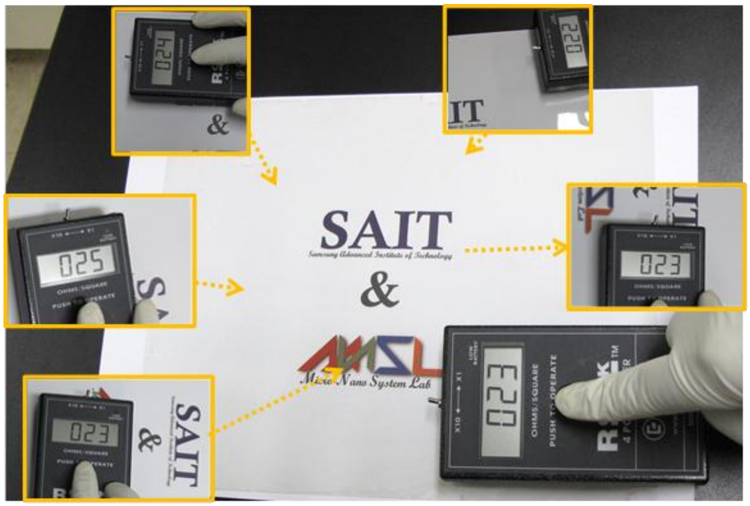

Figure S10. Visual demonstration of uniform sheet resistance of the CA-Ag NP/Ag NW TCF (25×20 cm^2) after the protective layer coating. The logos used in the figure were reproduced with permission from Samsung electronics and Sungkyunkwan University.

References

s1 Zhou, H., Wang, X., Yu, P., Chen, X. & Mao, L. Sensitive and selective voltammetric measurement of Hg2+ by rational covalent functionalization of graphene oxide with cysteamine. *Analyst* **137**, 305-308 (2012).

s2 Menendez, G. O. *et al.* Self-assembly of thiolated cyanine aggregates on Au(111) and Au nanoparticle surfaces. *Nanoscale* **4**, 531-540 (2012).

s3 Aina, V. *et al.* Novel bio-conjugate materials: soybean peroxidase immobilized on bioactive glasses containing Au nanoparticles. *J. Mater. Chem* **21**, 10970-10981 (2011).

s4 Kundu, S. & Nithiyanantham, U. In situ formation of curcumin stabilized shape-selective Ag nanostructures in aqueous solution and their pronounced SERS activity. *RSC Adv.* **3**, 25278-25290 (2013).

s5 Rajesh, K. G., Vinayak, A. D., Gosavi, S. W., Rishi, B. S. & Suwarna, S. D. Catalytic activity of allamanda mediated phytosynthesized anisotropic gold nanoparticles. *Adv. Nat. Sci: Nanosci. Nanotechnol.* **4**, 045005 (2013).

s6 Hu, L., Kim, H. S., Lee, J.-Y., Peumans, P. & Cui, Y. Scalable Coating and Properties of Transparent, Flexible, Silver Nanowire Electrodes. *ACS Nano* **4**, 2955-2963 (2010).

s7 Araki, T. *et al.* Low haze transparent electrodes and highly conducting air dried films with ultra-long silver nanowires synthesized by one-step polyol method. *Nano Res.* **7**, 236-245 (2014).

s8 Moon, I. K. *et al.* 2D Graphene Oxide Nanosheets as an Adhesive Over-Coating Layer for Flexible Transparent Conductive Electrodes. *Scientific Rep.* **3**, 1112 (2013).

s9 Song, M. *et al.* Highly Efficient and Bendable Organic Solar Cells with Solution-Processed Silver Nanowire Electrodes. *Adv. Funct. Mater.* **23**, 4177-4184 (2013).

s10 Kim, T. *et al.* Electrostatic Spray Deposition of Highly Transparent Silver Nanowire Electrode on Flexible Substrate. *ACS Appl. Mater. Interfaces* **5**, 788-794 (2013).

s11 Preston, C., Xu, Y., Han, X., Munday, J. & Hu, L. Optical haze of transparent and conductive silver nanowire films. *Nano Res.* **6**, 461-468 (2013).
